# Eye, Ear, Nose, and Throat

**Published:** 1895-04

**Authors:** 


### EYE, EAR, NOSE, AND THROAT.

EDITED BY DUNBAR ROY, A.B., M.D.

### IMPROVEMENT OF DEAF-MUTISM BY ACOUSTIC GYMNASTICS.

Such is the subject of an article by Dr. M. A. Goldstein, of St. Louis, in the last issue of the *Archives for Otology,* which to me is practical in its bearing, and perhaps so far-reaching in its result, that I deem it of interest to bring the subject before the profession at large.

According to Dr. Goldstein the proposition to attempt the treatmeant of deaf-mutism by the influence of aural gymnastics is by no means new.

Beck (*Ohrenheilkunde,* 1827, *p.* 7) notes the recommendation of Bewus in 1743 to treat deafness by sound conduction. From this time up to the present similar methods were used and recommended with varying success.

The treatment has again been actively revived by Professor Urbantschitsch, of Vienna, under whom the present writer worked some two years ago in that most delightful Austrian capital, and whose scientific scholarly attainments, coupled with exceptional charm of manner, will never be forgotten by his many pupils.

"Several years ago," writes Professor Urbantschitsch, "I obtained by systematic aural gymnastics surprising results in the case of a deaf mute boy presented to me for treatment. Primarily the patient was able to hear only loudly spoken vowels and single syllables; after one year's treatment he was able to understand complete sentences spoken in a moderately loud voice, and finally the ordinary methods of oral instruction were made possible by the steady improvement of the patient."

The past year Professor Urbantschitsch has instituted a daily class in aural gymnastics for the instruction of a large number of deaf mutes, and has obtained results of the most gratifying character. This group of children, who in previous examinations had been declared hopelessly deaf and ready for entrance to deaf mute asylums, has, since the adoption of methodical aural gymnastics, recovered not only their capacity of hearing *vowels*, but in the course of one year's practice have been able to hear and repeat *whole sentences*. Many of these cases at the first examination gave indications of absolute deafness, demonstrable by tuning-forks of different pitch and through air and bone conduction and most intense sounds applied to the ears; even the use of ear-trumpets, tubes and similar devices developed no sound perception." The writer of the article closes with these sentences:

"Such gratifying results reached while this system is still in its infancy, and in such comparatively short time, speak volumes. The introduction of a system which gives promise of so beneficial an aid in training and instruction of deaf mutes, should be granted a fair and patient trial in every deaf mute institution, and by every aural specialist in America. The Volta Bureau of Washington has, as I understand, received Professor Urbantschitsch's communications of this system, and is stimulating investigations as to its efficiency.

"Every medical man in general, and aurists in particular, should give the system of instruction pursued in our various institutions for deaf and dumb a careful study, so as to be able to recommend understandingly to parents and friends of the unfortunate members of society the course of training to be pursued best calculated to result in greatest advancement."

---

### NECROSIS OF THE LABYRINTH.

The above is the subject of an article read before the Medical and Chirurgical Faculty of Maryland and, later, published in the *New York Medical Journal*, by Dr. Harry Friedenwald, of Baltimore. The reported case is interesting, especially to the aurist of much clinical experience. The patient was a child four years old who, six months previous, had suffered with an attack of scarlatina, followed by a purulent discharge from both ears. Under injections of a boric acid solution, the right ear recovered, but the left continued to discharge. After cleansing the left ear thoroughly of a thick, creamy pus, a large polypus was found at the inner extremity of the canal, which was removed. The next day the patient had a chill, which was followed by a rise of temperature for several days, indicating retention of pus; was suffering with quite severe pain. The discharge not ceasing under the use of medicaments, Dr. Friedenwald opened the mastoid, finding an abscess, which was thoroughly opened and scraped. However, the discharge continued, a polypus again appeared in the middle ear, which was removed, and a fistulous track there found thoroughly scraped. The discharge still continuing and there being objective evidence of necrosis leading from the middle ear, Kuster's operation was performed. This consists in chiselling away a portion of the posterior and superior wall of the external canal, so as to expose the tympanum and attic thoroughly to view. This was done, and all necrosed tissue scraped away; but, in spite of all, the otorrhea returned. This latter was kept free from odor by boric acid and irrigations. Four years later a sequestrum found its way into the external canal, which was removed, the fistula closed, and since then there has been no discharge. The sequestrum was examined, and found to be a portion of a semi-circular canal. The patient at no time suffered with vertigo, a symptom, however, which, according to Bezold, has not been frequently observed.

Cases such as the one reported by Dr. Friedenwald are by no means infrequent—I mean, of course, only the persistent discharge due to a necrosis of some portion of the bony walls of the tympanum, and when they do fall into the hands of the aurist he is often to be pitied, since, as a rule, they are not cases upon which reputations can be the most frequently made. Often the discharge persists, even after two or three most radical operations and when the operator is perfectly sure that he has removed every semblance of necrotic tissue. The subject of curing discharges from the middle ear, is indeed a vexed one with the otologist and a condition which frequently causes the most worry in the specialist's hand.

One feature of this case which is worthy of mention is that the dressings with iodoform gauze produced quite a severe cutaneous eczema—so much so, that another dressing had to be substituted. That idiosyncrasy, for such it must be termed, is by no means infrequent. I have a patient now under treatment upon whom a most severe "weeping" eczema of the auditory canal was produced through packings of iodoform gauze in the canal. The discharge from the eczema was so profuse that the cotton in the ear was saturated four or five times during the day. For a while, I was puzzled to know how there could be so much discharge from the ear when the tympanic cavity looked so dry and healthy. I stopped the iodoform packings, painted the canal with a saturated watery solution of ichthyol, and in two days the canal and cotton were perfectly dry. In the use of iodoform gauze, physicians should always remember this peculiarity, and watch particularly any signs of an eczematous condition.

---

### A FURTHER OBSERVATION UPON THE USE OF TRICHLORACETIC ACID.

An article under this title has occurred in the January, 1894, issue of the *Monatsschrift für Ohrenheilkunde*, by Dr. Stanilaus von Stein, of Moscow, Russia, which is so practical that I shall reproduce in English, the main points brought out for the benefit of those whose privilege it has not been to read the article in the original.

Trichloracetic acid has, of late, been largely used as a chemical cauterant in treating affections of the nose and throat, and in fact with some it has almost entirely superseded chromic acid, which for so long a time held sway. It has been found to cauterize the mucous membrane deeper without spreading so much upon the surface, and the eschar which is left is found to be very easily detachable.

Von Stein opens his article with this sentence: "The peculiar caustic and astringent properties of trichloracetic acid for mucous membranes, which I have first (1889) called attention to, in an exhaustive article, since then have been confirmed by other observers. Some other good therapeutic peculiarities of this acid, which I have had occasion to demonstrate in the last two years, up to this time have scarcely or even not at all, been brought before the profession."

Here are some of the affections in which the acid is found beneficial:

1. In acute rhinitis, von Stein injects one-half teaspoonful of a warm one-tenth per cent. solution, three or four times in each nasal cavity, and finds that immediately after there is an abundant outflow of secretion soon followed by a subjective relief. He remarks that since he has used this "prophylactic treatment," he has seldom noticed consecutive ear inflammation in those patients who are usually afflicted every autumn and spring.

2. Weak trichloracetic acid solutions (one-half to one per cent.) used for some time in cases of simple atrophic rhinitis, not infrequently produce such a hypertrophy of the mucous membrane that one must resort to cauterization in order to produce free breathing. (?)

3. In true ozæna he uses a five-tenths to ten per cent. solution, and finds that it is a specific for destroying the odor. He begins with weak solutions and gradually increases them, applying the solution by means of cotton on the end of a probe, rubbing the solution thoroughly in every crack and crevice until every portion of crusts are removed and the parts touched. He does this first, two or three times daily, and then at longer intervals.

4. The author has used with success, the touching of the scar made by the galvano-cautery, with a saturated solution of the acid, by means of which there is never any reaction from the cautery, and it forms a white eschar which closely adheres to the mucous membrane for a few days, and then comes away leaving the membrane smooth and healthy looking. He has used this procedure since 1890 in all wounds of the nasal mucous membrane with most gratifying success.

5. The trichloracetic acid has this especial advantage in the nose, that by its use there is never any adhesive inflammation between closely situated particles, as sometimes follows in the use of chromic acid or the galvano-cautery, and for that reason the acid can be applied no matter how great the stenosis is.

6. In the pharynx, inflammation can be treated with equal success as in the nose. The author holds that tonsils can be greatly reduced in size by the use of these acid solutions. The crystals can be used for destroying the tonsillar crypts.

7. The application of this acid solution in the ear is painful, and must only be used on small granulations.

For applying the acid solutions in the nose, the author uses specula of different sizes, very long, reed-like. Those supplied by the instrument-makers in this country are unsatisfactory, so I have had Meyrowitz, of New York, to make me a set according to my own idea, which have proven very satisfactory.

